# Performance Analysis of NB-IoT Uplink in Low Earth Orbit Non-Terrestrial Networks †

**DOI:** 10.3390/s22187097

**Published:** 2022-09-19

**Authors:** Min-Gyu Kim, Han-Shin Jo

**Affiliations:** Department of Electronic Engineering, Hanbat National University, Daejeon 34158, Korea

**Keywords:** wireless communications, Internet of Things, non-terrestrial networks, doppler shift, NB-IoT, LEO satellite

## Abstract

The 3rd Generation Partnership Project (3GPP) narrowband Internet of Things (NB-IoT) over non-terrestrial networks (NTN) is the most promising candidate technology supporting 5G massive machine-type communication. Compared to geostationary earth orbit, low earth orbit (LEO) satellite communication has the advantage of low propagation loss, but suffers from high Doppler shift. The 3GPP proposes Doppler shift pre-compensation for each beam region of the satellite. However, user equipment farther from the beam center has significant residual Doppler shifts even after pre-compensation, which degrades link performance. This study proposes residual Doppler shift compensation by adding demodulation reference signal symbols and reducing satellite beam coverage. The block error rate (*BLER*) data are obtained using link-level simulation with the proposed technique. Since the communication time provided by a single LEO satellite moving fast is short, many LEO satellites are necessary for seamless 24-h communication. Therefore, with the *BLER* data, we analyze the link budget for actual three-dimensional orbits with a maximum of 162 LEO satellites. We finally investigate the effect of the proposed technique on performance metrics such as the per-day total service time and maximum persistent service time, considering the number of satellites and the satellite spacing. The results show that a more prolonged and continuous communication service is possible with significantly fewer satellites using the proposed technique.

## 1. Introduction

### 1.1. Research Background

The Internet of Things (IoT) market is expected to grow at an average annual rate of 30% [1]. In particular, the narrowband Internet of Things (NB-IoT) has excellent potential for development as a 5G IoT standard technology. The NB-IoT currently connects 100,000 devices and must improve its performance to meet the requirement for 5G communication [2,3]. Improvements in satellite communications are currently attracting attention. Satellites offer extensive coverage and are not affected by terrain obstacles [4]. The 3rd Generation Partnership Project (3GPP) is considering new radio (NR)-based non-terrestrial networks (NTN) for 5G activation and expansion. The 3GPP conducted a study on this topic in Release 15 [5], and a further study regarding IoT and enhanced machine type communication (eMTC) support for NTN was conducted in Release 17 [6,7]. The satellites discussed by the 3GPP include geostationary earth orbit (GEO) and low earth orbit (LEO) satellites. Satellites in LEO have the advantage of a shorter propagation delay than those in GEO; therefore, research on LEO satellites is being conducted actively. In particular, interest in LEO satellites has increased with the advent of Falcon 9, a reusable space launcher developed by SpaceX [8,9]. In conventional satellite communications, ground stations use parabolic antennas. However, since a parabolic antenna has a large volume, research on beamforming through a phased array antenna is being conducted [10,11,12].

Two problems must be addressed for LEO satellites to communicate with the NB-IoT. The first is a high Doppler shift. Unlike GEO satellites, LEO satellites orbit the Earth at high speeds when viewed from the Earth, causing high Doppler shifts to occur when communicating with them. This increases the carrier frequency offset, making it difficult for the receiver’s channel estimation [13,14,15]. Traditional LEO satellite communications involve a single communication link with the gateway. The global navigation satellite system (GNSS) determines the satellite’s position and calculates the Doppler shift, making compensation possible [16,17,18].

However, it is not easy to apply these methods to NB-IoT uplinks that use single carrier frequency division multiple access (SC-FDMA) [15,19]. SC-FDMA is a technology that bundles multiple carriers together. Because the bundled carrier has a single frequency but combines signals with different Doppler shifts, compensating for the individual Doppler shifts is challenging.

The second problem in data rate reduction is due to the long round-trip time (*RTT*). Among current NTN target performance scenarios, IoT connectivity requires a data rate of 10 kbps or more [20]. Still, it is challenging to meet the requirement because of the current long *RTT*. In 3GPP Release 17 [7], there is no consensus on a solution to improve throughput due to the battery life problem caused by GNSS. Therefore, this study shows the achievable throughput in the scenarios specified.

### 1.2. Related Work

The 3GPP considers common Doppler shift pre-compensation to solve the high Doppler shift [21]. This method offsets the same frequency for the received signal from all devices within the satellite beam coverage. Here, the frequency is estimated using the signal from the coverage center. Thus, the user equipment (UE) at the coverage edge suffers from a large residual Doppler shift even after the common Doppler shift compensation. The receiver must compensate for this to enable communication in the beam coverage. Currently, the residual Doppler shift compensation for NB-IoT uplinks ranges up to approximately 950 Hz [22,23].

The 3GPP proposes residual Doppler shift values for each satellite altitude and beam diameter. Table 1 summarizes the maximum Doppler shift and the residual Doppler shift after pre-compensation when an LEO satellite is at an altitude of 600 km [21]. The smallest residual Doppler shift they specify is 1.05 ppm for a beam diameter of 50 km, which amounts to 2100 Hz for the center frequency of 2 GHz. Therefore, additional solutions are needed to counterbalance the residual Doppler shift in NB-IoT receivers.

Two approaches have been studied for residual Doppler shift compensation mainly. The first approach is reducing the beam coverage of an LEO satellite. The authors of [24,25] solved the issue of Doppler shift compensation using the first approach. Kodheli et al. [24] addressed the maximum residual Doppler shift of LEO satellites with resource allocation. The system model assumes that an LEO satellite is at an altitude of 1000 km and has a beam diameter of 200 km. The beam coverage is divided into ten areas to satisfy the 950 Hz compensation range of the NB-IoT receiver. As a result, the maximum residual Doppler shift in one region decreases, making it possible to compensate for. Conti et al. [25] described the residual Doppler shift as an equation after compensating for the common Doppler shift. After that, the maximum beam coverage size of the satellite is proposed for each altitude of the satellite.

The second approach is increasing the compensation range for the Doppler shift of the NB-IoT receiver. There are two methods to accomplish this in OFDM-based communication systems [5,15,21]. The first method is to increase the subcarrier spacing (SCS). Currently, the maximum SCS for NB-IoT is 15 kHz. The Doppler shift compensation ranges are up to approximately 950 Hz, obtained by multiplying the SCS and frequency offset [13]. Although SCS expansion increases the compensation range [22,23], it also increases the bandwidth. It is not suitable for an NB-IoT receiver with a narrow bandwidth.

The second method involves adding the channel estimation symbols. In the NB-IoT standard, these are called the demodulation reference signal (*DMRS*) symbols. They are arranged at regular symbol intervals, with the property that the narrower the gap, the more accurate the channel estimation becomes. In the NB-IoT, one *DMRS* symbol is placed per slot. Therefore, placing two or three *DMRS* symbols per slot results in a narrower spacing and, thus, more accurate channel estimation [13].

*DMRS* symbol addition is also used in vehicle-to-everything (V2X) research. For vehicles, because of their high speed, a Doppler shift occurs. A *DMRS* symbol is added to solve this, which increases the receiver’s Doppler shift compensation range [26]. In addition, [27] analyzed the performance according to the number of *DMRS* symbols in the NR physical uplink control channel (PUCCH). When the UE speed is 500 km/h, adding *DMRS* symbols shows a better block error rate (*BLER*). In my previous study, which solved the Doppler shift by adding *DMRS* symbols, only *BLER* performance analysis was performed according to the number of iterations [28].

In previous studies, beam coverage size reduction is a proposed method to solve the residual Doppler shift problem. However, it does not address link performance degradation issues. This study proposes a minimum beam coverage size that can compensate for the residual Doppler shift calculated through 3D satellite orbits. In addition, link performance is improved by adding *DMRS* symbols, and the results are analyzed by defining various performance indicators.

### 1.3. Contributions and Organization

**Figure 1:**
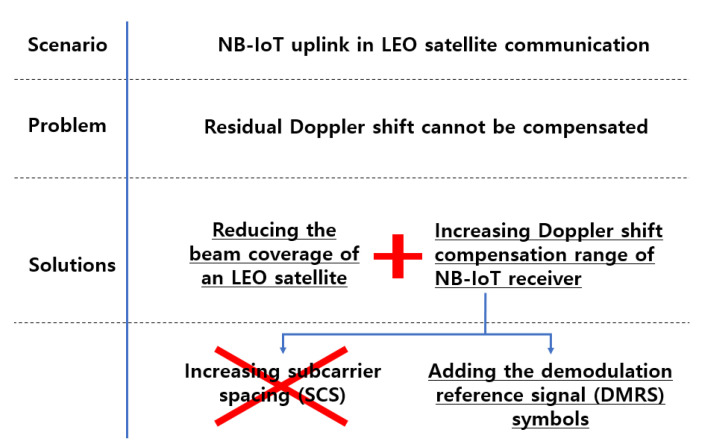
Problems and solutions for the NB-IoT uplink in NTN.

Figure 1 lists the problems and proposed resolutions for the NB-IoT uplink in LEO satellite communication. In this paper, we apply the underlined solution in Figure 1. Beam coverage reduction is proposed to address the residual Doppler shift. This study defines the relationship between the beam coverage size and the residual Doppler shift. Afterward, the maximum beam coverage size that the NB-IoT receiver can compensate for is calculated through 3D satellite orbit simulation. Even with the proposed beam coverage size, a residual Doppler shift exists, which degrades the link performance. In this study, we suggest adding *DMRS* symbols to improve link performance. To the best of our knowledge, previous studies have not implemented simulations using actual satellite orbits in the NB-IoT NTN scenario.

This study improved link performance by adding *DMRS* symbols to reduce the minimum number of satellites required to service NB-IoT NTN. Because LEO satellites are fast-moving, the time to communicate with NB-IoT is short. Therefore, it is necessary to increase communication time by deploying many satellites. However, as the number of satellites increases, the service cost increases, so information on the minimum number of satellites is required. In this study, STARLINK satellites are implemented through MATLAB to find the minimum number of satellites. Satellite orbits are implemented with two-line element (TLE) files [29]. The performance of *DMRS* symbol addition is verified through link-level simulation and link budget analysis. Link-level simulations show signal-to-noise ratio (SNR) and throughput results that satisfy *BLER* ≤ 0.1 [30]. Link budget analysis analyzes the results in four metrics by calculating the link margin during the day. The proposed multiple *DMRS* symbol shows that the same performance can be achieved using fewer satellites than a single *DMRS* symbol. This performance analysis method can be utilized as technical data for designing the LEO satellite communication system.

The remainder of this paper is organized as follows. Section 2 describes the system model. The NTN architecture and NB-IoT uplink transmission method are explained, and the channel model is described. Section 3 describes the residual Doppler shift problem and the link performance problem. A solution to the residual Doppler shift problem is presented in Section 4. Section 5 describes the evaluation metrics for performance analysis. Section 6 provides link-level and link budget simulation implementation and the numerical results. In Section 7, the conclusion and future work directions are presented.

## 2. System Model

### 2.1. NTN Architecture

**Figure 2:**
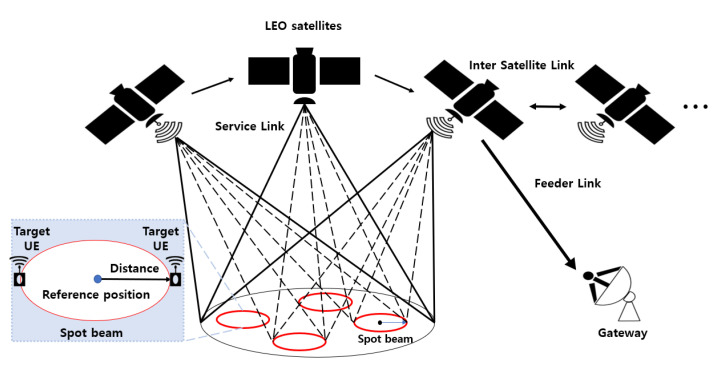
Architecture of the NB-IoT NTN.

Figure 2 shows the architecture of the NB-IoT NTN with service and feeder links. In this study, we apply the LEO-based Earth fixed cell scenario, where NTN provides a fixed service cell for a specific location on Earth for a particular amount of time. In addition to the LEO-based Earth-fixed cell scenario, 3GPP has presented an LEO-based Earth-mobile cell scenario, in which NTN provides a cell moving in satellite orbit [21]. The reasons for applying the LEO-based Earth fixed cell scenario in this study are as follows. (i) The Earth-fixed cell scenario is more straightforward than the Earth-moving cell scenario, which requires frequent handovers; (ii) NB-IoT communication would be required at a specific time, such as a notification message transfer. Therefore, rather than finding a new satellite that can communicate each time, we decided that an LEO-based Earth-fixed cell scenario that can communicate with the same satellite every time is better.

In this study, the reference position is latitude $36.35080^{\circ }$ N, longitude $127.30122^{\circ }$ E. The satellite orbits are implemented through 3D satellite simulation. The orbit can be determined through the TLE file and in this study, the Starlink satellite TLE file is used [29]. In real Starlink satellites, the Ka- and Ku-bands are used [8]. We utilized only orbital information from the Starlink satellite’s TLE file because 3GPP NB-IoT NTN in the S-band is currently under research and has not yet been commercialized. Therefore, the study was conducted using the parameters presented in the 3GPP document, not the Starlink parameters [21]. We also adopted Starlink satellite orbits because they are currently the most known satellites and have a relatively large number of satellites and orbits compared to other commercial satellites, allowing us to experiment with various orbits. Satellite and UE parameters are summarized in Table 2 and Table 3.

We consider the following assumptions regarding the architecture of the NB-IoT NTN. (i) The target UE is located within the spot beam; (ii) the satellite can steer beams towards fixed points on earth using beamforming techniques; (iii) assuming that the feeder and the inter-satellite link are ideal, the service link performance is analyzed; (vi) a minimum elevation angle of 10 degrees is considered for the UE and the satellite.

### 2.2. NB-IoT Uplink Transmission Scheme

In 3GPP Release 13, a cellular-based NB-IoT standard to support low-cost, low-power UE is confirmed. NB-IoT is a long-distance IoT communication technology that uses the long-term evolution (LTE) licensed band. LTE architecture is heavily reused in NB-IoT, including the numerologies, downlink orthogonal frequency-division multiple access (OFDMA), uplink single-carrier frequency-division multiple access (SC-FDMA), and other features. NB-IoT uplink channels include a narrowband physical uplink shared channel (NPUSCH) and a narrowband physical random access channel (NPRACH) [19]. This system model considers only the NPUSCH.

**Figure 3:**
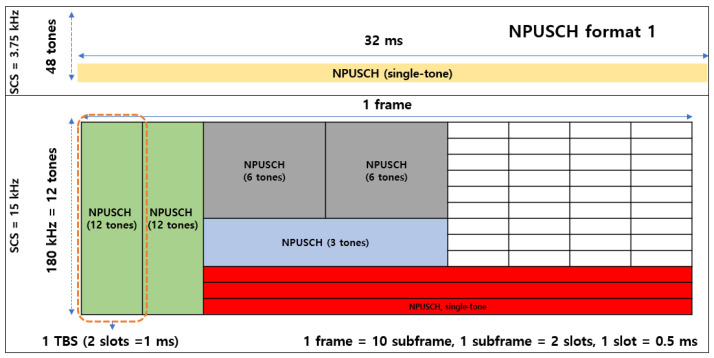
NB-IoT uplink frame structure (NPUSCH format 1).

**Figure 4:**
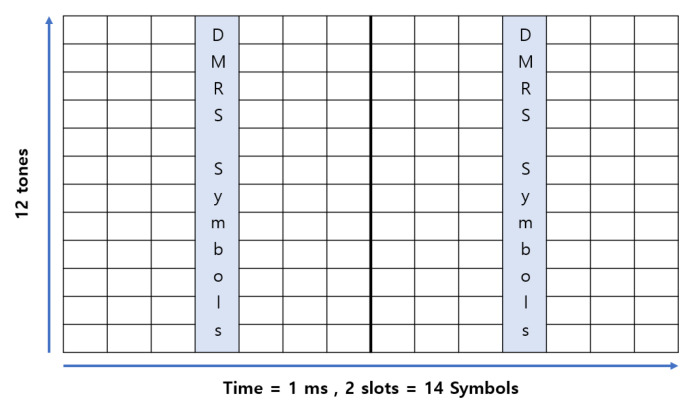
NB-IoT resource grid (1 slot = 0.5 ms, 1 tone = 15 kHz).

The NB-IoT SCS supports frequencies of 15 kHz and 3.75 kHz, setting bandwidths up to 180 kHz. The NB-IoT adds a resource unit (RU) concept to manage resources efficiently. One RU is the transport block size (*TBS*), which is defined by setting the number of tones and slots. Figure 3 shows a configurable transport block (TB) in an NPUSCH. In this study, the *TBS* is set by defining 12 tones and two slots as one RU. Figure 4 depicts the NB-IoT resource grid: an enlarged view of a TB configured with one *DMRS* symbol per slot. The NPUSCH format determines the number of *DMRS* symbols. Two NPUSCH formats are defined in [19,31]. Format 1 is used for general NB-IoT data transmission and has one *DMRS* symbol per slot. Format 2 uses a single tone and specifies three *DMRS* symbols per slot. In this study, NPUSCH Format 1 is selected.

HARQ is a hybrid type of error control method for forward error correction (FEC) and automatic repeat request (ARQ) methods [31,32]. FEC is an error correction technique that transmits a message by adding a redundancy bit and corrects an error when the data is lost. ARQ sends an acknowledgment (ACK) if there is no error when receiving data. The receiver sends a negative acknowledgment (NACK) if an error occurs to request retransmission.

HARQ is a hybrid method that combines the advantages of both methods. Incremental redundancy HARQ (IR-HARQ), currently used in NB-IoT, checks for errors every time one TB is transmitted. If an error occurs, a retransmission is requested by sending a NACK [33]. IR-HARQ transmits new information that includes an updated redundancy version (RV) value each time for retransmission. RV can be viewed as basic information, and additional bits are transmitted to estimate the data.

In LTE, the RV is divided into {0,2,3,1}, allowing four repetitions. The RV is divided and sent because the receiver stores the received data in a buffer before sending the NACK and compares it with the retransmitted data to correct the error. Unlike LTE, NB-IoT is divided into {0,2}. Instead, it corrects data errors by allowing an increased $N_{Rep}$. In the NB-IoT uplink, data transmission is possible by increasing $N_{Rep}$ up to 128 [34]. However, because NTN has a long *RTT*, the data rate decreases as $N_{Rep}$ increases. Therefore, in this study, the maximum analyzed value of $N_{Rep}$ is 2. $N_{Rep}$ is the number of retransmissions, including the first transmission. For example, if $N_{Rep}$ is 2, it is a value of one-time retransmission.

**Figure 5:**
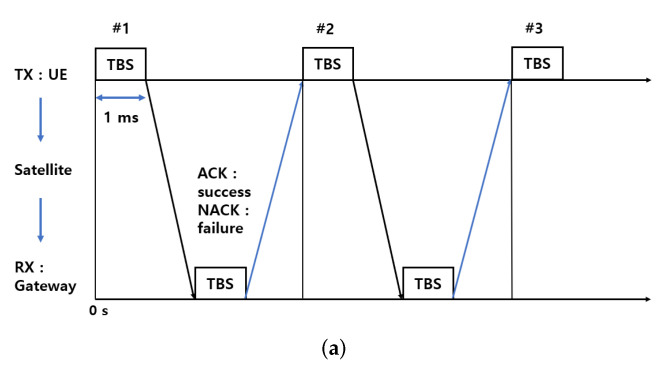

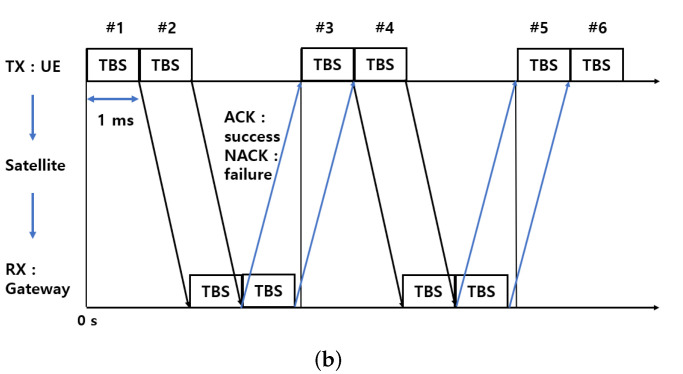
Comparison of the number of HARQ processes. (**a**) One HARQ process, (**b**) Two HARQ processes.

The HARQ process is a technique used in a system using HARQ, and increasing the number of HARQ processes improves the throughput of a system with a long *RTT*. The *RTT* of the satellite set in this study is about 25.77 ms. Currently, in the 3GPP, the maximum number of HARQ processes of the NB-IoT NTN is proposed to be two [7,34,35]. Figure 5 illustrates the effect of varying the number of HARQ processes. Figure 5a is a communication method with 1 HARQ process. When the number of HARQ processes is one, 1 TB (1 ms) is transmitted during *RTT* and not sent for 24.77 ms. Therefore, there is a delay until the subsequent TB is transmitted. Figure 5b is a communication method with 2 HARQ processes. If the number of HARQ processes is two, 2 TBs are transmitted in 25.77 ms and the throughput is doubled compared to using a single HARQ process. Increasing the number of HARQ processes raises the problem of growing memory buffer size and decreasing battery life. Therefore, the increase in HARQ processes is not considered in the current NB-IoT NTN system [7]. This study analyzes the throughput results by implementing a simulation including long *RTT*.

### 2.3. Channel Model

**Figure 6:**
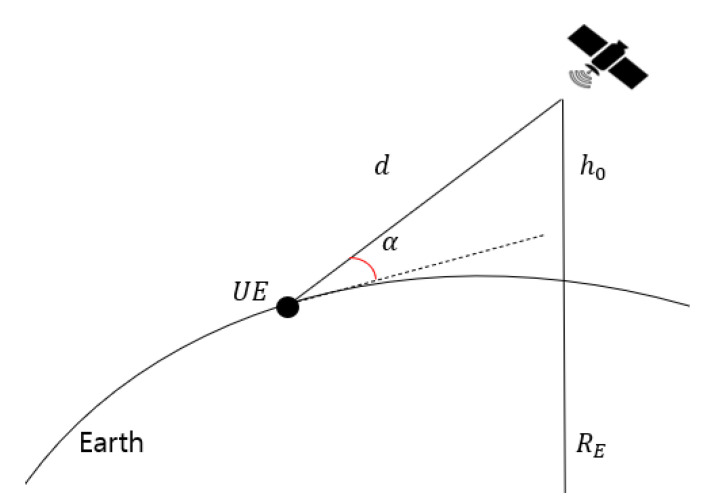
Slanted range *d* between a satellite and UE determined by $R_{E},h_{0}$, and α.

**Figure 7:**
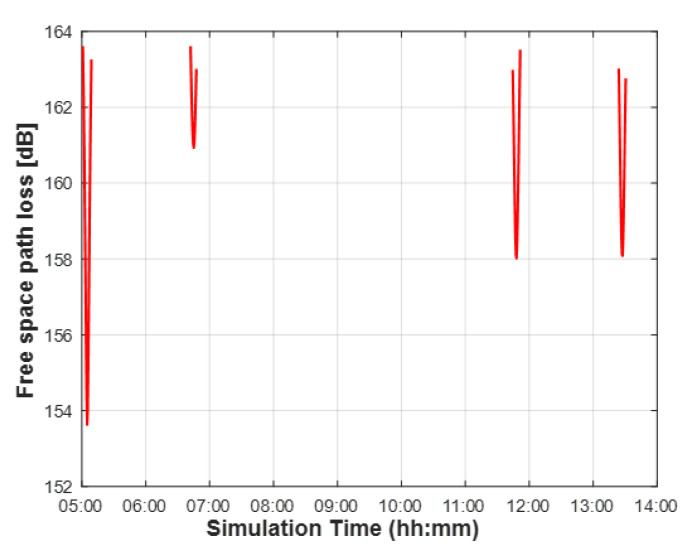
Free space path loss between the reference position (latitude $36.35080^{\circ }$ N, longitude $127.30122^{\circ }$ E) and a satellite receiver over simulation time based on the actual satellite orbit data on 22 February 2022.

The theoretical free-space path loss is given by

(1) $$PL_{FS}=32.45+20\log_{10}f_{c}+20\log_{10}d,$$

where $f_{c}$ is the center frequency in MHz and *d* is the distance in km. The distance between the satellite and the UE is shown in Figure 6, and *d* is expressed as follows [5]:

(2) $$d=\sqrt{R_{E}^{2}\sin\alpha^{2}+h_{0}^{2}+2h_{0}R_{E}}-R_{E}\sin\alpha ,$$

where $R_{E}$ (6371 km) is the Earth’s radius, α (>$10^{\circ }$) is the elevation angle, and $h_{0}$ is the altitude of an LEO satellite. This study calculates the distance between the satellite and the UE in one day with a sample time of 30 s. Table 4 summarizes the available communication time in the set system model. Figure 7 shows the free space path loss for an elevation angle of more than 10 degrees.

The path loss can be calculated as follows:

(3) $$PL=PL_{FS}+PL_{A}+PL_{SM}+PL_{SL}+PL_{AD},$$

where $PL_{FS}$ is the free space path loss, $PL_{A}$ is atmospheric loss, $PL_{SM}$ is the shadowing margin, $PL_{SL}$ is scintillation loss, and $PL_{AD}$ is additional loss [21].

**Figure 8:**
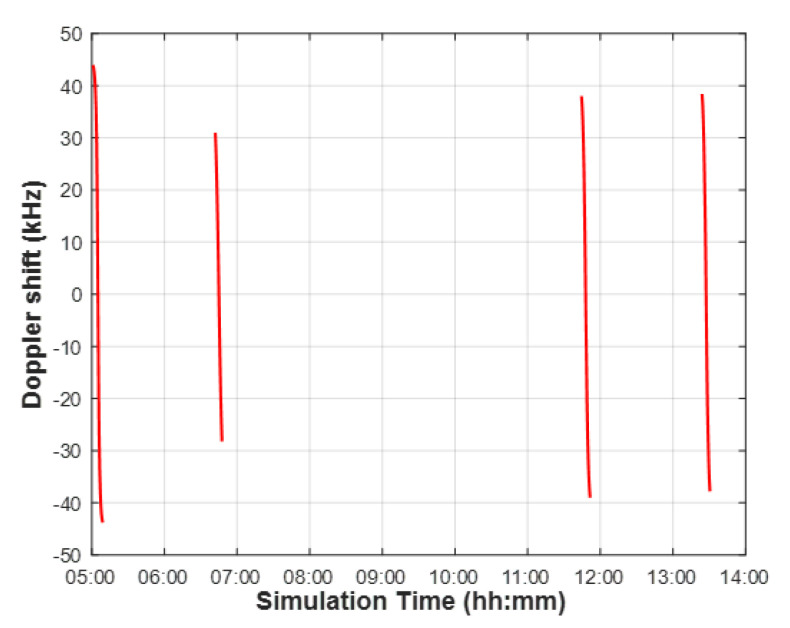
Doppler shift between the reference position (latitude $36.35080^{\circ }$ N, longitude $127.30122^{\circ }$ E) and a satellite receiver over a simulation time based on the actual satellite orbit data on 22 February 2022.

As an LEO satellite link is affected by a high Doppler shift, it must also be considered in the channel model. The Doppler shift at the LEO satellite receiver can be calculated as follows [15]:

(4) $$f_{d}=f_{c}\times \frac{v\cos\alpha }{c},f_{d,max}^{\left(1\right)}=f_{c}\times \frac{v}{c},$$

where *v* is the receiver speed, *c* is the light speed, and α is the angle between the transmitter and receiver. The maximum Doppler shift of the LEO satellites considered in this study is calculated through simulation. Figure 8 shows a time-varying Doppler shift between the reference position and a satellite receiver moving along with the actual orbit data. NB-IoT uplink communication is possible only by the satellite receiver compensating for the simulated Doppler shift. The 3GPP proposes pre-compensation to compensate for Doppler shift with the UEs deployed within a satellite beam coverage. The maximum residual Doppler shift with the UEs after pre-compensation is calculated by

(5) $$f_{residual}\left[Hz\right]=f_{reference}-f_{edge},$$

where $f_{reference}$ is the common Doppler shift that is equal to the Doppler shift with the reference position and $f_{edge}$ is the Doppler shift with the UE at the edge of beam coverage. Therefore, the residual Doppler shift of all the UEs are less than or equal to $f_{residual}$. From Equation (5), we can find that the maximum residual Doppler shift depends on the beam size. This is also observed in Table 1, where the Doppler shift is presented with a unit of ppm that can be converted to Hz by

(6) $$f_{d}\left[Hz\right]=\frac{f_{d}\left[ppm\right]\times f_{c}}{10^{6}}.$$

In addition to the path loss and Doppler shift model, we present the fading channel model for NB-IoT uplink NTN. The 3GPP studied fading channel models ranging from 0.5 to 100 GHz in TR 38.901 [36]. Among the fading channel models, the tapped delay line (TDL)-D model in an outdoor environment with a line of sight is applied to this study.

## 3. Problem Statement

### 3.1. Residual Doppler Shift

In LEO satellite communications, the problem of residual Doppler shift compensation must be addressed. In 3GPP, residual Doppler shift values after pre-compensation are tabulated. The parameters required for this study are given in Table 1, and we try to compensate for the residual Doppler shift value of 1.05 ppm [21]. Currently, the maximum Doppler shift an NB-IoT receiver can compensate for is around 950 Hz [22,23]. In this section, we formulate the residual Doppler shift compensation problem of NB-IoT NTN by calculating the Doppler shift compensation range through the formula.

The range of the Doppler shift compensation depends on the frequency offset, which is calculated as follows [13]:

(7) $$\hat{\epsilon }=\frac{N_{FFT}\times \hat{\theta }}{2\pi \times L},$$

where $N_{FFT}$ is the fast Fourier transform (FFT) size (128) and $\hat{\theta }$ is the phase difference between channel estimation symbols. *L* is the number of time samples between channel estimation symbols. Here, the channel estimation symbol is expressed as a *DMRS* symbol in the NB-IoT. *L* is calculated as follows [13]:

(8) $$L=\sum_{i=1}^{n}\left(L_{cp}\left(i\right)+N_{FFT}\right),$$

where *n* is the number of symbols between the *DMRS* symbols and $L_{cp}\left(i\right)$ is the number of cyclic prefix (CP) time samples of the *i*-th symbol. CP is a guard interval that removes interference between previous symbols [37]. In the NB-IoT, there are 10 CP time samples in the first symbol and 9 in the rest. As a result, *L* for the NB-IoT is 960-time samples.

The NB-IoT uplink modulation technique is quadrature phase shift keying (QPSK). Therefore, the maximum phase difference between *DMRS* symbols is π. Using Equation (7), $\hat{\epsilon }$ is 1/15. The maximum Doppler shift for which the NB-IoT receiver can compensate is calculated as follows [13]:

(9) $$f_{d,max}^{\left(2\right)}=SCS\times \hat{\epsilon },$$

where *SCS* is 15 kHz. The value of $f_{d,max}^{\left(2\right)}$ is 1 kHz based on Equation (9). This value is the theoretical maximum possible Doppler shift compensation. Considering this, 3GPP uses Equation (4) to say that compensation is possible up to about 950 Hz when *v* is 500 km/h. The residual Doppler shift to be compensated for is about 2100 Hz using Equation (6). As a result, current NB-IoT receivers cannot compensate for the residual Doppler shift of 2100 Hz. Therefore, additional solutions are needed to compensate for the residual Doppler shift.

### 3.2. Link Performance

NB-IoT is a low-power system with a small EIRP [38]. Therefore, link performance is essential for communicating with LEO satellites farther than the base station. LEO satellites have a Doppler shift, which further degrades the link performance of NB-IoT. Terrestrial NB-IoT improves link performance through retransmission using HARQ [39]. However, NB-IoT NTN has difficulty tolerating many retransmissions due to the long *RTT* of LEO satellites. In particular, for LEO satellites, the communication time varies depending on the orbit and the communication time is short and variable. Therefore, service providers are trying to establish links through multiple LEO satellites to solve the LEO satellite communication problem. Although an analysis of the minimum number of satellites is required to establish a link, there are not many studies for NB-IoT NTN [40,41]. Therefore, a new method to improve the link performance in NB-IoT NTN is required, and an analysis of the number of satellites, applying satellite orbit, is required. In addition, since NB-IoT periodically transmits data such as notification messages, it is also necessary to analyze the communication time for varying numbers of LEO satellites.

## 4. Proposed Solution

### 4.1. Reduction in Beam Coverage

Section 3.1 calculated the NB-IoT receiver range. Currently, this study’s residual Doppler shift that needs to be compensated for is 2100 Hz [21]. This study proposes methods for solving the problem. The proposed method sets the beam diameter to be smaller than the beam diameter suggested by 3GPP. Although this method is simple, the benefits of satellites are lost as the coverage size becomes smaller. Therefore, proper size is essential. This study expresses the relationship between beam coverage and residual Doppler shift as an equation. In addition, beam coverage that satisfies 950 Hz or lower is calculated through satellite orbit simulation.

Equation (5) described in Section 2.3 is expressed in detail as follows:

(10) $$\begin{array}{cc} f_{residual} & =f_{reference}-f_{edge}\\ \; & =f_{c}\times \frac{v}{c}\times \left|\left(\cos\theta_{1}-\cos\theta_{2}\right)\right|, \end{array}$$

where $\theta_{1}$ is the angle between the satellite and the reference position. $\theta_{2}$ is the angle between the satellite and the target UE. The maximum residual Doppler shift occurs when the angle formed with the reference position is 90 degrees. The equation for calculating the beam coverage is as follows:

(11) $$\begin{array}{cc} f_{max} & \geq f_{c}\times \frac{v}{c}\times \left|\left(-\cos\theta_{2}\right)\right|,\;\theta_{1}=90^{\circ }\\ \; & \geq f_{c}\times \frac{v}{c}\times \frac{R}{\sqrt{R^{2}+h_{0}^{2}}}\\ R & \leq \sqrt{\frac{h_{0}^{2}}{{\frac{v\times f_{c}}{c\times f_{max}}}^{2}-1}}, \end{array}$$

where *R* is the radius of the beam coverage, $h_{0}$ is the satellite’s altitude, and $f_{max}$ is the maximum residual Doppler shift. Equation (11) is calculated assuming that the Earth and the satellite are in two parallel lines.

**Figure 9:**
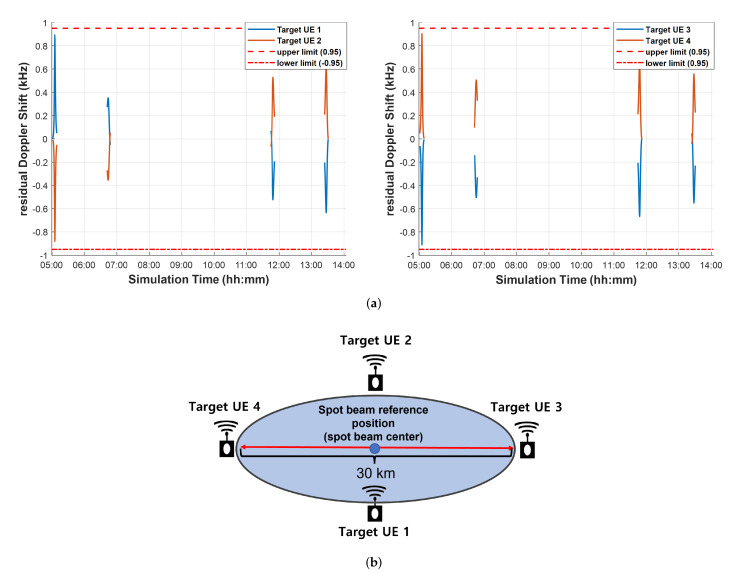
Doppler shift and maximum coverage size compensable by NB−IoT receivers. (**a**) Residual Doppler shift, (**b**) Target position.

This paper calculates the maximum coverage size the NB-IoT receiver can compensate for through simulation. Figure 9 shows the simulation results. Figure 9a shows the results of the residual Doppler shift in each target UE. During the simulation time, the residual Doppler shift is satisfied [0.95 −0.95], and the beam diameter is 30 km. Figure 1b shows the target position applied in Figure 9a.

### 4.2. Addition of the DMRS Symbol

By reducing the beam diameter, the residual Doppler shift of 2100 Hz is reduced to 950 Hz. However, this method also has its problems. The required SNR increases because residual Doppler shift is still present, resulting in lower link performance than without the Doppler shift. Cellular NB-IoT improves link performance through excessive repetition. However, if the NB-IoT is repeated excessively, the time delay increases and the data rate decreases. Therefore, we try to solve this problem by adding more *DMRS* symbols instead of increasing the number of repetitions. The *DMRS* symbol addition per slot suppresses link performance degradation even with residual Doppler shifts.

**Figure 10:**
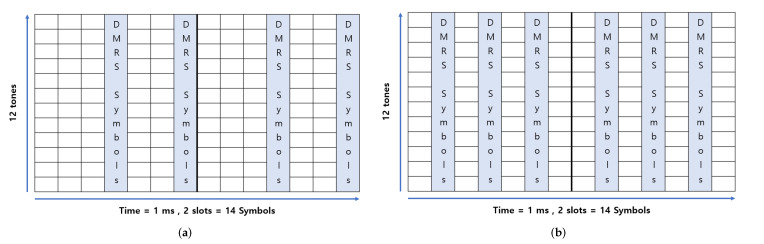
Proposed NB-IoT resource grid. (**a**) Two *DMRS* symbols per slot, (**b**) Three *DMRS* symbols per slot.

We suggest increasing the number of *DMRS* symbols ($N_{dmrs}$) per slot to two or three. Figure 10a shows the NB-IoT resource grid with two *DMRS* symbols per slot, where the *DMRS* symbols are arranged in the fourth and seventh symbols. This arrangement is because the CP length of the first symbol is longer than that of the other symbols. Therefore, placing the *DMRS* symbol on the seventh symbol gives better results in terms of link performance. Figure 10b shows a resource grid of three *DMRS* symbols per slot. Adding this shows that the performance is suitable only when the number of *DMRS* symbols is appropriately increased.

## 5. Performance Analysis

In this section, evaluation metrics used for performance analysis are presented. In link-level simulation, *BLER* and throughput are used. Furthermore, link margin, impossible communication time, and total communication available time are defined for link budget calculation and analysis.

### 5.1. Block Error Rate (BLER)

*BLER*, used for link-level performance evaluation, is calculated as follows:

(12) $$\begin{array}{c} BLER=\frac{failure\;TBs}{total\;TBs}, \end{array}$$

where *total TBS* is the total number of *TBS* sent and *failure* *TBS* is the number of *TBS* that failed to transfer. This study denotes an SNR that satisfies *BLER* ≤0.1 as $SNR_{required}$ [30].

### 5.2. Throughput

Throughput, considering *RTT* of LEO satellites, is calculated as follows:

(13) $$Throughput\left(bps\right)=\frac{TBS}{RTT+TTI},$$

where *RTT* is the time taken for the signal sent by the UE to arrive at the gateway via the satellite, assuming 25.77 ms [21]. The transmission time interval (*TTI*) depends on the number of RUs, a unit for NPUSCH scheduling. *TBS* is the total number of transmitted bits and is calculated as follows:

(14) $$TBS\left(bits\right)=\left(T_{RE}-DMRS_{RE}\right)\times N_{BIT},$$

where $T_{RE}$ is the total number of resource elements (REs), $DMRS_{RE}$ is the total number of REs in the *DMRS* symbols, and $N_{BIT}$ is the number of bits per RE.

### 5.3. Link Margin

The link budget is typically calculated as follows [42,43]:

(15) $$\begin{array}{c} SNR_{received}\left(dB\right)=EIRP-\left(PL_{FSL}+PL_{A}+PL_{SM}+PL_{SL}\right)+\frac{G}{T}-k-B, \end{array}$$

where $SNR_{received}$ denotes the received SNR in dB for a receiver, EIRP is the effective isotropic radiated power, $\frac{G}{T}$ is the antenna-gain-to-noise-temperature, and *k* is the Boltzmann constant, which is −228.6. *B* is the bandwidth. Units are dBW for EIRP, dB/K for $\frac{G}{T}$, dBW/K/Hz for *k*, and dBHz for *B*. Therefore, the link margin is calculated as follows:

(16) $$\begin{array}{c} LM_{i}=SNR_{received}-SNR_{required}. \end{array}$$

This study calculates $LM_{i}$ every *i*-th sample time of 30 s through satellite orbit simulation for 24 h. Let us define an indicator function $1_{\left\{LM_{i}<0\right\}}$ = 1 if $LM_{i}$< 0 and zero otherwise. We assume a communication outage for the *i*-th sample time of 30 s corresponding to $LM_{i}$< 0. We define the per-day outage time ratio as follows:

(17) $$P_{OTR}\left(\%\right)=\frac{30\;sec\times \Sigma_{i}1_{\left\{LM_{i}<0\right\}}}{24\times 3600\;sec}\times 100.$$

Next, we define the per-day total service time in h with an indicator function $1_{\left\{LM_{i}\geq 0\right\}}$ = 1 if $LM_{i}$≥0, and zero otherwise as follows:

(18) $$T_{S}=\frac{30\;sec\times \Sigma_{i}1_{\left\{LM_{i}\geq 0\right\}}}{3600}.$$

We define the maximum persistent service time (*MPST*) in h as follows:

(19) $$MPST=\frac{30}{3600}\times max\left(i_{2}-i_{1}\right)\;such\;that\;LM_{i}\geq 0\;for\;all\;i\in \left[i_{1},i_{2}\right],$$

where, *i*, $i_{1}$, and $i_{2}$ are the time indices of sampling the link margin data. With a similar expression, we define the maximum persistent outage time (*MPOT*) in h as follows:

(20) $$MPOT=\frac{30}{3600}\times max\left(i_{2}-i_{1}\right)\;such\;that\;LM_{i}<0\;for\;all\;i\in \left[i_{1},i_{2}\right].$$

The metrics defined above are essential for IoT NTN. Furthermore, it is crucial to estimate the minimum number of satellites needed to meet the target value of the metrics, which are provided in the following section.

## 6. Numerical Results

### 6.1. Link-Level Simulation

This section provides simulation results of uplink NB-IoT performance in the LEO satellite communication system. We implement a link-level simulator for an NB-IoT NPUSCH. The 3GPP TDL-D channel model [36] is adopted and the maximum Doppler shift is set to 950 Hz. The simulator outputs the *BLER* and throughput results with the SNR. $N_{Rep}$ is set to 2. The two slots comprise one TB with a transmission time of 1 ms. The *TBS* is set to 120 bits [34]. The NB-IoT receiver checks for errors when it receives one TB. If an error occurs, retransmission is requested; otherwise, new data is requested. The simulation reports the *BLER* and throughput results when 5000 *TBS* have been transmitted. The link-level simulation parameters are summarized in Table 5.

**Figure 11:**
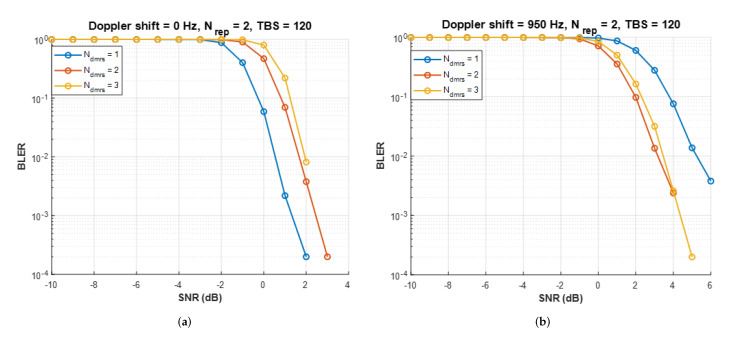
Uplink transmission *BLER* result for varying numbers of *DMRS* symbols. (**a**) Doppler shift = 0 Hz, (**b**) Doppler shift = 950 Hz.

Figure 11a shows *BLER* curves for zero residual Doppler shift. One *DMRS* symbol has the lowest required SNR for satisfying *BLER* ≤0.1 [30]. However, in Figure 11b, the link performance deteriorates due to the residual Doppler shift of 950 Hz. The distance between *DMRS* symbols determines the NB-IoT receiver Doppler shift compensation range; the more significant the Doppler shift, the greater the influence of the *DMRS* symbols. Therefore, in the residual Doppler shift calculated in this study, the performance is good at two *DMRS* symbols. Terrestrial network NB-IoT has improved link performance through many iterations. However, in NB-IoT NTN, many reiterations are impossible due to time delays. Therefore, *DMRS* symbol addition can be a practical solution to improve *BLER* performance.

**Figure 12:**
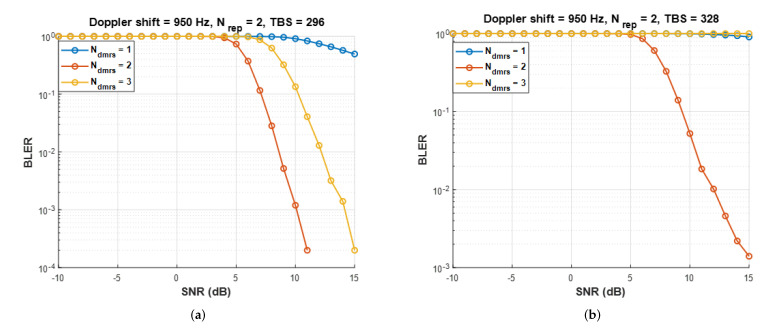
Uplink transmission *BLER* result according to *TBS* with various numbers of *DMRS* symbols. Doppler shift = 950 Hz, (**a**) *TBS* = 296 bit, (**b**) *TBS* = 328 bit.

Figure 12 shows BELR performance results according to *TBS*, illustrating the advantages and disadvantages of adding *DMRS* symbols instead of data symbols. In Figure 12a, when $N_{dmrs}=1$, a possible SNR satisfying *BLER* ≤0.1 cannot be obtained owing to an excessively high Doppler shift [30]. On the contrary, *DMRS* symbol addition can obtain a feasible SNR thanks to its improved Doppler compensation range. Despite increased overhead, it achieves a much higher data rate than $N_{dmrs}=1$. Figure 12b plots *BLER* curves when more bits (*TBS* = 328 bits) are sent than the maximum number of bits that can be sent with $N_{dmrs}=3$. Here, $N_{dmrs}=3$ yields the worst *BLER* because the overhead is too large to convey the transport block; rather, $N_{dmrs}=2$ shows the best *BLER*. In conclusion, the appropriate number of *DMRS* symbols should be selected according to the Doppler shift and *TBS*.

**Figure 13:**
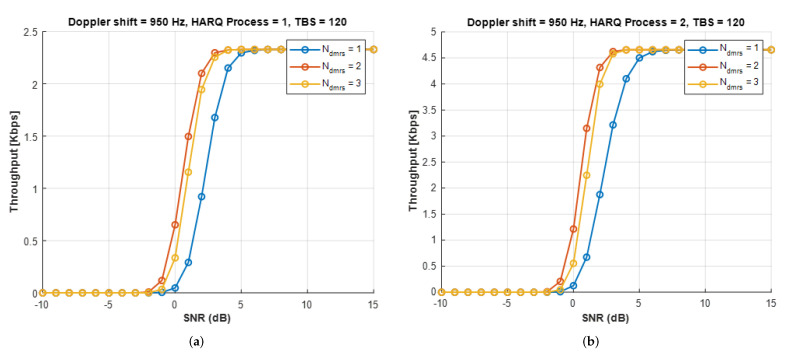
Uplink throughput for different numbers of *DMRS* symbols per slot with one or two HARQ processes. (**a**) One HARQ process, (**b**) Two HARQ processes.

Figure 13 plots throughput curves of NB-IoT NTN with $N_{Rep}$ = 2 for one and two HARQ processes. Compared to the terrestrial NB-IoT link, the NTN uplink transmission involves a much longer *RTT* of 25.77 ms. Therefore, in Figure 13a, the throughput of one HARQ process is limited to 2.3 kbps. Figure 13b shows that the maximum throughput doubles up to 4.6 kbps with the help of the two HARQ processes. Among current NTN target performance scenarios, IoT connectivity requires a data rate of 10 kbps or more [20]. Although increasing the number of HARQ processes can satisfy the requirement, it is not preferred due to the increase in the data buffer and the risk of shortening battery life. Therefore, it is necessary to analyze this issue in future studies.

### 6.2. Link Budget Simulation

The link budget simulation shows the results for one day using Equation (16). $SNR_{required}$ is the minimum SNR that satisfies *BLER* ≤0.1 and $SNR_{required}$ depends on $N_{dmrs}$. Figure 11b shows the minimum $SNR_{required}$ for different $N_{dmrs}$; 3.7 dB for $N_{dmrs}=1$, 2 dB for $N_{dmrs}=2$ and 2.3 dB for $N_{dmrs}=3$. $SNR_{received}$ is calculated through Equation (15) and the parameters used in the simulation are summarized in Table 6. The link budget simulation calculates $LM_{i}$ every 30 s through a 3D satellite orbit simulation implemented in MATLAB. The LEO satellite used in this study is a STARLINK satellite and the STARLINK satellite orbit is implemented using the TLE file. TLE references the North American Aerospace Defense Command (NORAD) [29]. To see the effect of adding *DMRS* symbols, we set the number of satellites and the number of satellite orbits as variables. The number of satellites is set from 1 to 27 per orbit, and simulations are performed in 1 and 6 orbits. This study assumes that a maximum of 27 satellites can be deployed in one orbit by setting the minimum interval for continuous communication of LEO satellites.

The inter-satellite spacing is set in two ways according to the arrangement method of the LEO satellites. In the first method, called fixed-spacing, the inter-satellite spacing does not change with the number of satellites. Inter-satellite spacing is set as the interval when there are 27 satellites equally spaced in one orbit. The second method, called variable-spacing, is that as the number of satellites increases, the spacing between satellites becomes narrower, and the spacing is the same as in the first method when there are 27 satellites. In other words, the satellites are uniformly filled around the perimeter of an orbit.

**Figure 14:**
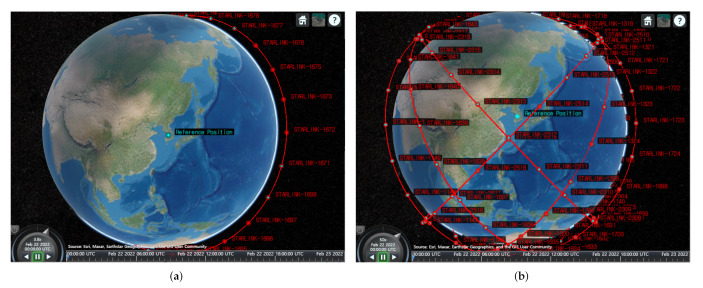
3D satellite orbits implemented in MATLAB [44]. (**a**) one orbit (27 satellites), (**b**) six orbits (162 satellites).

Figure 14 shows the implemented 3D satellite orbit. Figure 14a shows 27 satellites “STARLINK-1671”. The spacing between each satellite is the same, and as the number of satellites decreases, the spacing increases. Figure 14b shows 162 satellites “STARLINK-1671”, “STARLINK-1298”, “STARLINK-2498”, “STARLINK-1625”, “STARLINK-2299”, and “STARLINK-1139” [29]. The time set in the simulation is from 22 February 2022 to 23 February 2022, and all satellites have the same parameters.

**Figure 15:**
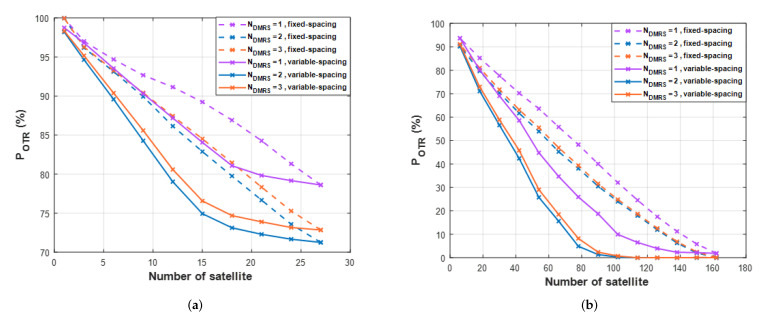
The per−day outage time ratio for different numbers of *DMRS* symbols per slot. (**a**) one orbit, (**b**) six orbits.

Figure 15 shows the $P_{OTR}$ result. The lower $P_{OTR}$ can be achieved using fewer satellites when using variable-spacing rather than fixed-spacing. Therefore, performance analysis is focused on variable-spacing. Figure 15a presents the result of one orbit. In one orbit, the simulation is implemented by adding three satellites each. Increasing the number of *DMRS* symbols improves the performance of $P_{OTR}$ by about 5%. However, in one orbit, even if the number of satellites is increased, it is more than 70%, so it is difficult to give a significant meaning. Therefore, in LEO satellite communication, the performance of one orbit is not good. Figure 15b presents the result of six orbits. At six orbits, the number of satellites is up to 162. The simulation is performed in increments of 12 total satellites (two satellites per orbit). First of all, when $N_{dmrs}=1$, the minimum $P_{OTR}$ is 1.87% and more orbits are needed to satisfy 0% $P_{OTR}$. On the other hand, at $N_{dmrs}=2,3$, the minimum $P_{OTR}$ is 0%, which shows good performance. The proposed method satisfies $P_{OTR}$ of 1% or less when using at least 102 satellites (17 satellites per orbit). The proposed *DMRS* symbol addition shows lower $P_{OTR}$ using fewer satellites.

**Figure 16:**
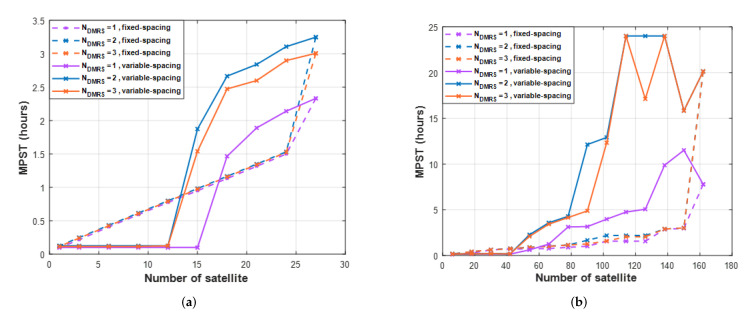
The maximum persistent service time (*MPST*) for different numbers of *DMRS* symbols per slot. (**a**) one orbit, (**b**) six orbits.

Figure 16 shows the maximum persistent service time (*MPST*) for the different numbers of *DMRS* symbols per slot. The number of *DMRS* symbols has little effect on the *MPST* performance of the fixed-spacing. However, it affects the *MPST* of the variable-spacing. In Figure 16a, the result of one orbit, the maximum *MPST* is 2.5 h for $N_{dmrs}=1$ and the maximum *MPST* for $N_{dmrs}=2,3$ are more than 3 h. In one orbit, there is little effect of adding *DMRS* symbols. Figure 16b shows the result of six orbits, which offers a large difference from one orbit. The maximum *MPST* is 12 h for $N_{dmrs}=1$ and 24 h for $N_{dmrs}=2,3$. In addition, the maximum *MPST* for $N_{dmrs}=2,3$ can be obtained using 114 satellites (19 satellites per orbit). On the other hand, the *MPST* for $N_{dmrs}=1$ cannot exceed 12 h, even for 162 satellites. As a result, the maximum *MPST* is obtained using fewer satellites.

The *MPST* for $N_{dmrs}=2,3$ increases as the number of satellites increases, and 114 satellites in operation achieve the maximum *MPST*. However, if the number of satellites increases by more than 114, *MPST* will be reduced. Satellite intervals narrow when the number of satellites increases, and if the spacing is too narrow, the performance decreases, depending on the communication situation. Therefore, research on LEO satellite orbit is required for more stable communication.

**Figure 17:**
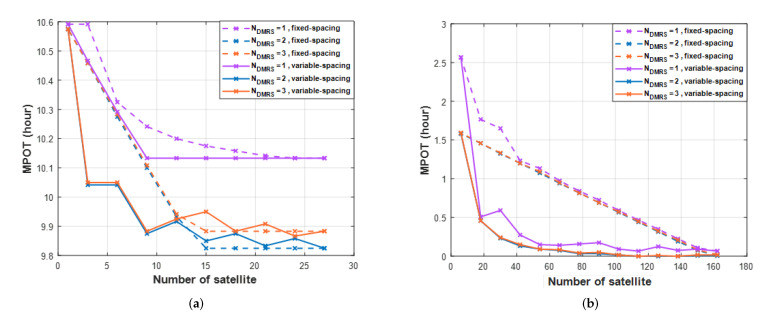
The maximum persistent outage time (*MPOT*) for different numbers of *DMRS* symbols per slot. (**a)** one orbit, (**b**) six orbits.

Figure 17 shows the maximum persistent outage time (*MPOT*). The *MPOT* analysis is based on variable-spacing because the performance is better when applying variable-spacing than fixed-spacing. In Figure 17a, the results of one orbit, the minimum *MPOT* is 10.12 h for $N_{dmrs}=1$, and the minimum *MPOT* for $N_{dmrs}=2,3$ are less than 9.9 h. As a result, in one orbit, it is difficult to see the performance difference of *MPOT* according to the proposed method. Figure 17b shows the results in six orbits. Compared to Figure 17a, *MPOT* shows better results. The minimum *MPOT* is 0.1 h for $N_{dmrs}=1$ and the minimum *MPOT* for $N_{dmrs}=2,3$ are zero h. Therefore, the proposed method obtains the minimum (zero h) *MPOT*. There is also a difference between fixed-spacing and variable-spacing. The variable-spacing allows using fewer satellites to achieve good performance. For example, the number of satellites to meet less than 0.1 h is 90 $N_{dmrs}=2,3$ and 162 in $N_{dmrs}=1$. Although the performance difference is not significant in the *MPOT*, it is possible to achieve similar performance using a smaller number of satellites.

**Figure 18:**
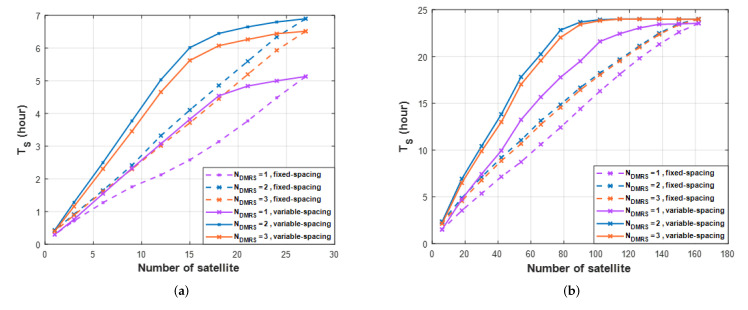
The per−day total service time for different numbers of *DMRS* symbols per slot. (**a**) one orbit, (**b**) six orbits.

Figure 18 presents the $T_{S}$ result. When applying variable-spacing, not fixed-spacing, one can use fewer satellites. For example, in Figure 18a, when $N_{dmrs}=2$, the number of satellites that satisfy $T_{S}>5$ h is 12 in variable-spacing and 18 in fixed-spacing. Variable-spacing can save about six satellites. Therefore, the performance according to $N_{dmrs}$ is analyzed in variable-spacing. In Figure 18a, the results of one orbit, the maximum $T_{S}$ is 5 h for $N_{dmrs}=1$ and the maximum $T_{S}$ for $N_{dmrs}=2,3$ are more than 6.5 h. Using the method suggested, one can communicate for 1.5 h longer. The time of 6.5 h is 27% out of 24 h, and 5 h is 20%, which is about a 7% improvement in performance. However, about 73% of the time one is still unable to communicate, so the impact is insignificant in one orbit. Figure 18b presents the $T_{S}$ result in six orbits. The maximum $T_{S}$ is 23.55 h for $N_{dmrs}=1$, and the maximum $T_{S}$ for $N_{dmrs}=2,3$ are 24 h. $T_{S}$ does not make a large difference, but the proposed method performs better with fewer satellites. For example, when $N_{dmrs}=2,3$, the maximum (24 h) $T_{S}$ is possible using 114 satellites, but when $N_{dmrs}=1$, the maximum $T_{S}$ is 23.55 h using 162 satellites. By adding *DMRS* symbols, one can achieve good performance with 48 fewer satellites. As a result of the analysis in this study, the addition of *DMRS* symbols shows that it can satisfy existing performance standards by using fewer satellites.

## 7. Conclusions

In this study, to improve NB-IoT coverage, we considered the NB-IoT NTN structure, which assumes that the LEO satellite is transparent. We formulated the residual Doppler shift compensation problem in LEO satellite communications. The residual Doppler shift is calculated by realizing the actual STARLINK satellite in 3D. We calculated the residual Doppler shift related to the beam coverage size. We proposed a beam diameter that fits the NB-IoT receiver compensation range. Since the NB-IoT device has a small EIRP, *DMRS* symbol addition is proposed to improve link performance. The proposed *DMRS* symbol addition avoids the degradation of link performance even in the presence of Doppler shift. These results are presented through link-level simulation, and then link performance results are analyzed through link budget analysis. A 3D satellite simulation is implemented through real satellite orbits. In this study, we analyzed results such as $P_{OTR}$, $T_{S}$, *MPST*, and *MPOT*. We observe that just one *DMRS* symbol addition remarkably saves 72 satellites to achieve 23.55 h of service time per day. The results can offer an interesting perspective for implementing cost-effective NB-IoT NTN. In future research, we could find ways to increase throughput, such as the HARQ process, which is not currently considered in NB-IoT.

## Figures and Tables

**Table 1:** Maximum Doppler shift and the residual Doppler shift after pre-compensation.

Parameter	Value
Frequency band	S-band (2 GHz)
Satellite altitude	600 [km]
Maximum Doppler shift	24 [ppm]
Residual Doppler shift	1.05 [ppm] for 50 km beam diameter
after	1.88 [ppm] for 90 km beam diameter
pre-compensation	15.82 [ppm] for 1000 km beam diameter

**Table 2:** LEO satellite parameters.

Parameter	Value
Satellite altitude	552 [km]
Satellite effective isotropic radiated power (EIRP)	34 [dBW/MHz]
Satellite antenna gain	30 [dBi]
Equivalent satellite antenna aperture	2 [m]
Antenna gain-to-noise-temperature (G/T)	1.1 [dB/K]

**Table 3:** UE parameters.

Parameter	Value
Frequency band ($f_{c}$)	S-band (2 GHz)
Antenna type and configuration	(1,1,2) with omnidirectional antenna element
Polarization	Linear: $+/-45^{\circ }X-pol$
Antenna temperature	290 [K]
Noise figure	7 [dB]
Transmit power	200 [mW]
Antenna gain	0 [dBi]

**Table 4:** Communication available time with STARLINK-1698 on 22 February 2022.

Source	Target	Start Time	End Time	Duration [s]
Reference Position	STARLINK-1698	05:01:30	05:09:00	450
Reference Position	STARLINK-1698	06:42:30	06:47:30	300
Reference Position	STARLINK-1698	11:44:30	11:51:00	390
Reference Position	STARLINK-1698	13:24:00	13:30:30	390

**Table 5:** Link-level simulation parameters.

Parameter	Value
FFT size	128
SCS	15 [kHz]
Multiple access	SC-FDMA
Modulation	QPSK
Channel coding	1/3 Turbo code
*TBS*	120 bits
Fading channel model	3GPP TDL-D
Residual Doppler shift	0, 950 [Hz]
$N_{Rep}$	2
Bandwidth	180 [kHz]

**Table 6:** Link budget simulation parameters.

Parameter	Value	
Transmit power	−6.99 dBW	200 mW ->10log(0.2)
Transmit antenna gain	0 dBi	Antenna gain
EIRP (effective isotropic radiated power)	−6.99 dBW	Transmit power + antenna gain
Atmospheric loss	0.07 dB	[21]
Shadow fading margin	3.00 dB	
Scintillation loss	2.20 dB	
Polarization loss	0.00 dB	
Additional losses	0.00 dB	
Boltzmann’s constant [*k*]	−228.6 dBW/K/Hz	
Bandwidth [B]	52.55 dBHz	

## Data Availability

Not applicable.
